# Neuroprotective effects of donepezil against Aβ_25-35_-induced neurotoxicity

**DOI:** 10.1186/s40001-022-00862-1

**Published:** 2022-10-28

**Authors:** Bu-Lang Gao, Ning-Ning Che, Xue Li, Chun-Feng Ren

**Affiliations:** 1 Department of Medical Research, Shijiazhuang People’s Hospital, 9 Fangbei Road, Shijiazhuang, 050011 Hebei Province China; 2 Neurology, Henan Provincial People’s Hospital, School of Clinical Medicine, Henan University, Zhengzhou, 450003 China; 3 Department of Laboratory Test, First Affiliated Hospital, Zhengzhou University, Zhengzhou, 450003 China

**Keywords:** Donepezil, Beta amyloid 25–35, Alzheimer’s disease, Protein kinase C, Neuroprotection

## Abstract

**Purpose:**

The purpose of this study was to investigate the neuroprotective effect of donepezil against β-amyloid_25-35_ (Aβ_25-35_)-induced neurotoxicity and the possible mechanism.

**Methods:**

PC12 cells were conventionally cultured. Serial concentrations of Aβ_25-35_ and donepezil (0, 0.5, 1, 5, 10, 20 and 50 μmol/L) were added to the PC12 cells, and 3-(4,5-dimethylthiazol-2-yl)-2,5-diphenyl-2H-tetrazolium bromide (MTT) staining was performed to detect the effects of these treatments on PC 12 viability. The PC 12 cells were pretreated with 1, 5, 10, 20 or 50 μmol/L donepezil two hours before 20 μmol/L Aβ_25-35_ was added to pretreatment groups A, B, C, D and E. Normal control group I and the 20 μmol/L Aβ_25-35_-treated group were selected. An MTT assay was used to detect PC12 cell viability, and the level of lactate dehydrogenase (LDH) was determined. PC12 cells were pretreated with 10 μmol/L GF109203X (a protein kinase C [PKC] antagonist) 30 min before 10 μmol/L donepezil was added to pretreatment group F, and normal control group II, the 10 μmol/L GF109203X-treated group and the 10 μmol/L donepezil-treated group were chosen. The expression of phosphorylation-PKC (P-PKC) and its major substrate phosphorylated myristoylated alanine-rich protein C kinase substrate (P-MARCKS) was measured by Western blotting. The effects of donepezil on the subcellular distribution of the PKCα and PKCε isoforms were detected by immunofluorescence staining.

**Results:**

Treatment with Aβ_25-35_ (5, 10, 20 or 50 μmol/L) for 24 h significantly (*P* < 0.05) decreased PC 12 cell viability in a dose-dependent manner. Compared with the PC12 cells in the control group, those in the 20 μmol/L Aβ_25-35_-treated group exhibited lower viability but higher LDH release. Compared with the 20 μmol/L Aβ_25–35_-treated group, pretreatment groups B, C, D and E exhibited significantly (*P* < 0.05) increased cell viability but significantly (*P* < 0.05) decreased LDH release. Western blotting demonstrated that compared with control, 10 μmol/L donepezil promoted PKC and MARCKS phosphorylation and that the expression of P-PKC and P-MARCKS in pretreatment group F was significantly (*P* < 0.05) lower than that in the donepezil-treated group. Immunofluorescence staining revealed that the PKCα and PKCε isoforms were located mainly in the cytoplasm of PC12 control cells, whereas donepezil increased the expression of the PKCα and PKCε isoforms in the membrane fraction. The Western blot results showed that donepezil altered the subcellular distribution of the PKCα and PKCε isoforms by decreasing their expression in the cytosolic fraction but increasing their expression in the membrane fraction.

**Conclusion:**

Donepezil can antagonize Aβ_25–350_-induced neurotoxicity in PC 12 cells, and PKC activation may account for the neuroprotective effect of donepezil.

## Introduction

As a neurodegenerative disease characterized by progressive loss of memory and cognitive impairment, Alzheimer’s disease (AD) is the most common form of dementia in the elderly [1, 2]. There are two pathological hallmarks of AD: extracellular deposits of amyloid-β (Aβ) protein and intracellular phosphorylated tau protein [3–6]. Abnormal extracellular deposition of the Aβ peptide results in the formation of senile plaques in the hippocampus, which is the pathological feature of AD associated with neurodegeneration [7]. The Aβ protein may trigger the hyperphosphorylation of the tau protein and cause impairment of axonal transport and destabilization of microtubules, leading to neuronal apoptosis. This neurotoxicity of the Aβ protein is one of the most important mechanisms of AD, and inhibition of the neurotoxicity of this protein is thus one approach for treating this disease. Currently, the predominant agents for treating AD are acetylcholinesterase inhibitors, which can enhance cholinergic neurotransmission in the synaptic cleft by inhibiting acetylcholine degeneration [8]. As a classical inhibitor of acetylcholinesterase approved by the FDA, donepezil can provide symptomatic relief and neuroprotection against Aβ toxicity in cell and animal models [9–11] and halt progressive atrophy in the brains of AD patients [12]. However, the specific pharmaceutical mechanism of donepezil is not clear, especially regarding its effects on neuroprotection against exogenous Aβ; however, some research has indicated that donepezil may be able to alter the metabolism of the amyloid precursor protein, leading to decreased secretion of Aβ [13]. This study investigated the effect of donepezil on rat pheochromocytoma cells (PC 12) exposed to Aβ_25-35_ and the possible mechanism.

## Materials and methods

### Reagents

Donepezil was provided by Eisai China Inc. (Suzhou, China). Aβ_25-35_, dimethyl sulfoxide (DMSO), a mouse monoclonal anti-β-actin antibody and 3-(4,5-dimethylthiazol-2-yl)-2,5-diphenyl-2H-tetrazolium bromide (MTT) were provided by Sigma-Aldrich (St. Louis, MO, USA). The protein kinase C (PKC) inhibitor GF109203X was provided by Calbiochem (San Diego, CA, USA). A rabbit polyclonal anti-phospho-PKC (P-PKC) antibody and a rabbit polyclonal anti-phospho-myristoylated alanine-rich C kinase (MARCKS) antibody were provided by Cell Signaling Technology (Beverly, MA, USA). Mouse monoclonal anti-PKCα and PKCε antibodies were provided by Santa Cruz Biotechnology (Santa Cruz, CA, USA). A Dulbecco’s modified Eagle medium (DMEM), foetal bovine serum (FBS), penicillin, streptomycin and pancreatin-EDTA (ethylene diamine tetraacetic acid) mixture was provided by Gibco (Carlsbad, CA, USA). RIPA cell lysis buffer was provided by Shenneng Bocai (Shanghai, China). A BCA protein assay kit was provided by Pierce (Pierce Biotechnology, Rockford, IL, USA). An ECL Western Blotting Detection kit was provided by Amersham-Pharmacia Biotech (GE Healthcare, Little Chalfont, UK).

### Cell culture

PC12 cells were routinely plated into 100 mm culture dishes in DMEM containing 10% foetal bovine serum, 1% streptomycin and 1% penicillin. The cells were cultured in 100% humidity and 5% CO_2_ at 37 °C. When the cells reached 80% cell fusion, the medium was discarded, and the cells were lysed with pancreatin for passage. Cells in logarithmic growth were taken for experiments.

### MTT assay

The MTT assay was used to assess cell viability. The cells were plated in a 96-well plate at a density of 1 × 10^4^ cells per well. Twenty-four hours later, donepezil or Aβ_25-35_ at 0, 0.5, 1, 5, 10, 20 or 50 μmol was added to the cells, with six wells for each concentration. At the same time, medium without cells was used as the blank control. Twenty-four hours after treatment, the medium was replaced, and 180 μL of medium and 20 μL of 5 g/L MTT were added to each well for further incubation for 4 h. Then, the medium was discarded, and 200 μL of DMSO was added to each well for incubation for 30 min at 37 °C. The absorption of the cells at 570 nm was evaluated and compared with that of the cells in the control group to determine the relative viability of the cells.

### Effect of donepezil pretreatment on cell viability

The cells were plated at a density of 1 × 10^4^ cells per well in a 96-well plate and were divided 24 h later into the normal control group, the Aβ_25-35_-treated group, and the Aβ_25-35_ plus 1, 5, 10, 20, or 50 μmol donepezil-treated groups. For the Aβ_25-35_ plus donepezil-treated groups, donepezil was added at different concentrations and then 20 μmol/L Aβ_25-35_ was added two hours later for further incubation for 24 h. Then, cell viability was evaluated by the MTT assay (as described above).

### Lactate dehydrogenase (LDH) release

The manufacturer’s protocol was used to assay LDH released from damaged cells. The treatment and classification of the groups were the same as those described above. The supernatant was collected from each group, and protein inhibitors were added on ice. Then, 120 μL of supernatant was transferred to an enzyme-labelling plate. After the reaction, working fluid was added and incubated for 30 min at room temperature, and the absorbance at 500 nm was measured.

### Western blotting for P-PKC and P-MARCKS expression

The cells were inoculated at a density of 10^5^ cells/mL in a 10-cm culture dish. The cells were divided into the normal control group, the donepezil-treated group, the GF109203X plus donepezil-treated group and the GF109203X-treated group. Thirty minutes after 10 μmol/L GF109203X was added to the GF109203X plus donepezil-treated group and the GF109203X-treated group, 10 μmol/L donepezil was added to the donepezil-treated group and the GF109203X plus donepezil-treated group, and an equivalent amount of solvent was added to the normal control group. After the protein concentration was measured in each group, 20 μg of protein was taken for sodium dodecyl sulphate (SDS)-polyacrylamide gel (PAGE) electrophoresis. Electrophoresis was performed at 100 v for 20 min and then at 150 v for 60 min. Then, the proteins were electrophoretically transferred to polyvinylidene difluoride membranes (Bio-Rad, Hercules, CA, USA), which were saturated with 5% non-fat milk and incubated with β-actin (1:10,000), phospho-PKC (1:1000) and P-MARCKS (1:1000) primary antibodies at 4 °C overnight. After washing with Tris-buffered saline (TBS) for 30 min, the membranes were incubated with horseradish peroxidase-conjugated anti-rabbit/mouse IgG secondary antibodies (1:1000) for one hour at room temperature. TBS was used to wash the membranes, and ABC compound was added at room temperature for 30 min. The signals were developed using an ECL Western Blotting Detection kit.

### Immunofluorescence for PKCα and PKCε expression

The cells were inoculated at a density of 10^5^ cells/mL in 6-well plates, and the cells were divided into the control and donepezil-treated groups. In the donepezil-treated group, 10 μmol/L donepezil was added after the cells were inoculated, while the inoculated cells of the normal group were not treated with donepezil. Two hours later, the medium was discarded, and PBS was used to wash the cells twice. Thirty minutes after 40 g/L paraformaldehyde fixation, PBS was used to wash the cells three times for 5 min each time. Then, the cells were treated with PBS containing 3% H_2_O_2_ and 0.1% Triton X-100 for 20 min at room temperature, and PBS was used to wash the cells. The cells were incubated with PBS containing 3% bovine serum albumin (BSA) for 30 min at room temperature, and a mouse monoclonal anti-PKCα (1:100) or anti-PKCε (1:100) antibody was added at 4 °C overnight. After washing with PBS, a FITC-conjugated anti-mouse IgG secondary antibody was added and incubated for 90 min. After washing with PBS three times, fluorescent glycerol was used for mounting, and the cells were observed under a microscope.

### PKC translocation

A PKC translocation assay was conducted according to the procedure described by Etcheberrigaray et al. with some modifications [14]. In brief, after cell lysis in lysis buffer (20 mM Tris–HCl (pH 7.5), 2 mM EDTA, 2 mM EGTA, 5 mM DTT, 0.32 M sucrose and 2 mM PMSF), the extracts were centrifuged at 12,000 × *g* for 20 min at 4 °C. The resulting supernatants were regarded as the cytosolic fractions. The pellets were incubated with the same lysis buffer, to which 1% Triton X-100 was added on ice for 45 min. After centrifugation at 12,000 × *g* for 20 min at 4 °C, the supernatants were collected for use as the membrane fractions. After BCA (bicinchoninic acid) quantification, 20 mg of protein mixed with 5 × loading buffer and 20 × reducing agent was boiled for 5 min and loaded onto a 7.5% SDS-PAGE gel. The following procedure was the same as that described above, except that the primary antibodies were mouse monoclonal anti-PKCα and anti-PKCε antibodies (1:1000 in BSA).

### Statistical analysis

SPSS 19.0 software (IBM, Chicago, IL, USA) was used for statistical analysis. The data are presented as the mean ± standard deviation (SD). Comparisons between multiple groups were performed using one-way ANOVA, and comparisons between two groups were performed using LSD *t*-test. *P* < 0.05 was considered statistically significant.

## Results

### Effects of Aβ_25-35_ and donepezil on cell viability

Treatment with Aβ_25-35_ at 5, 10, 20 or 50 μmol/L for 24 h significantly (*P* < 0.05) decreased the cell viability from 85.26 ± 8.03 to 40.84 ± 4.28, whereas donepezil had no significant effects on the cell viability at any of the concentrations (from 0.5 μmol/L to 50 μmol/L) (Table 1).

**Table 1:** Effects of Aβ_25-35_ and donepezil on cell viability (*n* = 6, mean ± SD)

Concentration (μmol)	Cell viability (Aβ_25-35_)	Cell viability (donepezil)
Control	100	100
0.5	97.53 ± 8.54	99.28 ± 6.76
1	85.26 ± 8.03	98.29 ± 8.78
5	78.94 ± 7.67*	97.94 ± 5.45
10	70.69 ± 7.32*	99.78 ± 5.74
20	56.65 ± 4.15*	97.48 ± 5.56
50	40.84 ± 4.28*	95.56 ± 7.85
*P*	0.00	0.23

*P < 0.05 compared with the control group

### Effects of donepezil on Aβ_25-35_-induced cell viability and LDH release

Compared with the normal control, 20 μmol/L Aβ_25-35_ significantly (P < 0.05) decreased cell viability but increased LDH release. Compared with the Aβ_25-35_, Aβ_25-35_ + 5, 10, 20 or 50 μmol donepezil significantly (*P* < 0.05) increased the cell viability from 57.35 ± 3.95 to 87.35 ± 7.42 but decreased LDH release from 164.57 ± 14.52 to 138.25 ± 5.93, indicating that 5–20 μmol donepezil antagonized the decrease in cell viability induced by 20 μmol Aβ_25-35_ (Table 2).

**Table 2:** Effects of donepezil on Aβ_25-35_-induced cell viability and LDH release (*n* = 6, mean ± SD)

Groups	Cell viability	LDH
Control	100	100
Aβ_25-35_	57.35 ± 3.95^*^	164.57 ± 14.52^*^
Aβ_25-35_ + 1 μmol donepezil	71.83 ± 6.01	152.63 ± 11.35
Aβ_25-35_ + 5 μmol donepezil	79.61 ± 7.72^**^	129.26 ± 11.56^**^
Aβ_25-35_ + 10 μmol donepezil	86.37 ± 7.45^**^	124.21 ± 7.44^**^
Aβ_25-35_ + 20 μmol donepezil	88.98 ± 9.36^**^	129.66 ± 5.27^**^
Aβ_25-35_ + 50 μmol donepezil	87.35 ± 7.42^**^	138.25 ± 5.93^**^
P	0.004	0.000

*P < 0.05 compared with the control group

**P < 0.05 compared with the Aβ_25-35_ group. The concentration of Aβ25-35 administered to each group was 20 μmol/L

### P-PKC and P-MARCKS expression

**Fig. 1:**
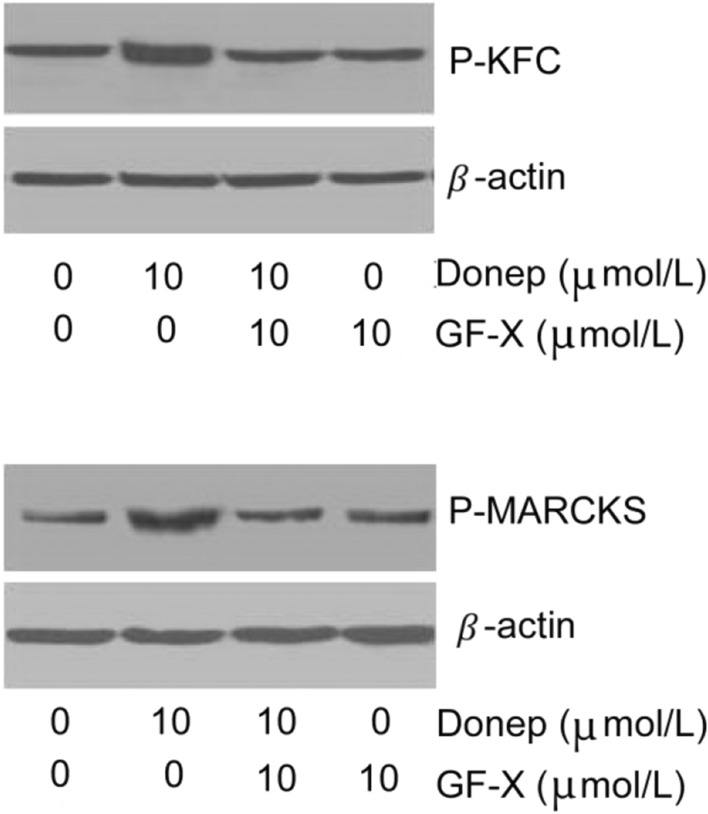
Effects of donepezil on PKC and MARCKS phosphorylation. **A** P-PKC. **B** P-MARCKS. 1. Normal control group; 2. Donepezil group; 3. GF109203X + donepezil group; 4. GF109203X group

Compared with that in the normal control group, P-PKC and P-MARCKS expression was significantly (*P* < 0.05) increased in the 10 μmol donepezil-treated group (158.86 ± 11.62 and 147.56 ± 12.05, respectively). Compared with that in the donepezil-treated group, P-PKC and P-MARCKS expression was significantly (*P* < 0.05) decreased in the GF109203X + donepezil-treated group (99.67 ± 10.36 and 99.64 ± 8.86, respectively, Fig. 1 and Table 3).

**Table 3:** P-PKC and P-MARCKS expression

Groups	P-PKC	P-MARCKS
Control	100	100
Donepezil	158.86 ± 11.62^*^	147.56 ± 12.05^*^
GF109203X + donepezil	99.67 ± 10.36^**^	99.64 ± 8.86^**^
GF109203X	95.47 ± 8.53	93.58 ± 7.36
P	0.001	0.015

*P-PKC* phosphorylated protein kinase C; *P-MARCKS* phospho-myristoylated alanine-rich C kinase substrate

*P < 0.05 compared with the control group, and **P < 0.05 compared with the donepezil group

### Effects of donepezil on PKC subtype distribution

**Fig. 2:**
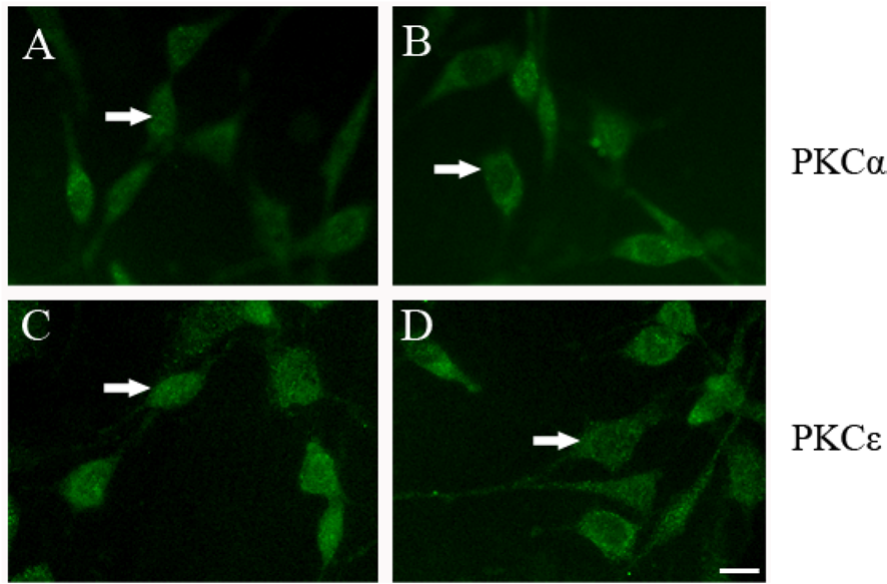
Effects of donepezil on the subcellular distribution of PKC isoforms(scale = 40 μm). **A** PKCα in the normal control group. **B** PKCα in the donepezil group with. **C** PKCε in the normal control group. D. PKCε in the donepezil group

**Fig. 3:**
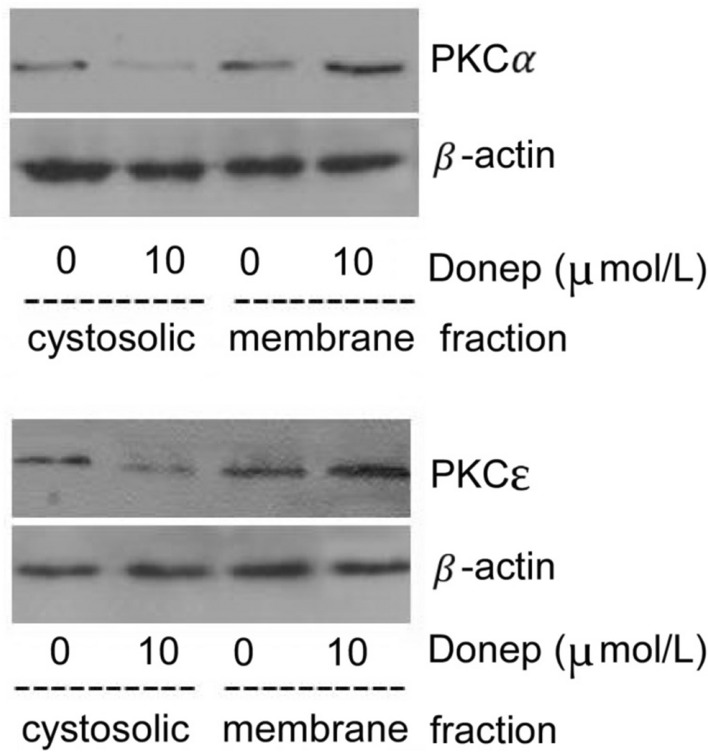
Effects of donepezil (Donep) on the subcellular distribution of the PKCα and PKCε isoforms. PC 12 cells were treated with vehicle alone or with 10 μm donepezil for 2 h. Cytosolic and membrane proteins were then extracted separately for Western blot analysis. Donepezil decreased the expression of PKCα (from 100 to 74.83 ± 9.41, *P* < 0.01) and PKCε (from 100 to 79.75 ± 8.16, *P* < 0.05) in the cytosolic fraction but increased their expression (PKCα from 100 to 130.89 ± 12.45, *P* < 0.01; PKCε: from 100 to 148.39 ± 15.58, *P* < 0.001) in the membrane fraction

Immunocytochemical fluorescence staining demonstrated that the PKCα and PKCε proteins were mostly evenly distributed in the cytoplasm in the normal control group but exhibited higher expression in the cytomembrane in the 10 μmol donepezil-treated group (Fig. 2). Additionally, the expression of the PKCα and PKCε isoforms in the cytosolic and membrane fractions was measured by Western blot separately to further confirm the subcellular distribution after donepezil treatment. The results showed that donepezil decreased the expression of PKCα (from 100 to 74.83 ± 9.41, *P* < 0.01) and PKCε (from 100 to 79.75 ± 8.16, *P* < 0.05) in the cytosolic fraction but increased their expression (PKCα: from 100 to 130.89 ± 12.45, *P* < 0.01; PKCε: from 100 to 148.39 ± 15.58, *P* < 0.001) in the membrane fraction (Fig. 3). This implies that donepezil can induce the translocation of the PKCα and PKCε isoforms from the cytosol to the membrane, indicating that donepezil administration can activate the PKC signalling pathway.

## Discussion

Our study demonstrated that the acetylcholinesterase inhibitor donepezil antagonizes the ability of Aβ_25-35_ to decrease cell viability. This protective effect of donepezil was partially offset by the PKC inhibitor GF109203X, indicating that the PKC pathway participates in the cellular protective effect of donepezil. Donepezil increased the expression of P-PKC and P-MARCKS and altered the distribution of PKCα and PKCε, suggesting that donepezil has an activating effect on PKC, which may be the mechanism by which donepezil protects against Aβ_25-35._

Twenty-four hours after treatment with 5–50 μmol/L Aβ_25-35_, the viability of PC 12 cells was decreased in a dose-dependent manner; however, donepezil at the same concentration did not significantly affect cell viability. In contrast, 1–50 μmol/L donepezil antagonized the neurotoxicity of 20 μmol Aβ_25-35_ at. Compared with the control, treatment with Aβ_25-35_ led to marked changes in PC 12 cells, including cell shrinkage, diminished cell bodies, reduced protrusions and decreased adherence ability. These cell changes were protective responses that resulted from exposure to foreign toxic substances, and pretreatment with donepezil significantly antagonized the toxic effects. However, the specific proteins that participate in the morphological changes of cells and the detailed mechanism remain to be explored.

PKC plays a very important regulatory role in the initiation and development of AD, and PKCα and PKCε are thought to be closely related to the nosogenesis of AD. Normally, PKC is located in the cytoplasm, and when it is activated, it is transported to the membrane serosa [15] through a process called protein trafficking. PKCε has also been found to modulate the activity of α secretase by participating in M receptor-mediated sAPPα secretion [16]. In the fibroblasts of AD patients, the function of PKC has been found to be lacking, leading to decreased responses of neurons to various growth factors and neurotransmitters; additionally, decreased PKC activity together with decreased trafficking ability of PKC subtypes is closely related to decreased memory and cognitive function [16, 17]. The downstream substrate of PKC, MARCKS, is expressed in the mouse brain and is associated with the spatial learning ability of animals. In senile plaques in AD, MARCKS coexists with PKC and Aβ, and the phosphorylation of MARCKS is considered to be a marker of PKC activation, which is associated with neuroprotection [18]. PKCε is often associated with preconditioning neuroprotection and mediates ischaemic tolerance by activating ERK in brain slices [19, 20]. PKCα has also been implicated in both cell survival and cell death signalling pathways and is activated in the rat brain upon ischaemic preconditioning [21–23]. The activation of PKC is neuroprotective and can increase neuronal strength against apoptosis and maintain cellular homeostasis through the phosphorylation of HSP27 [24, 25]. PKCε induces neuroprotection against ischaemia by regulating many pathways, including the phosphorylation of the mitochondria K + ATP channel [20], increased synaptosomal mitochondrial respiration [26] and the activation of the extracellular signal-regulated kinase (ERK) pathway [19], via N-methyl-d-aspartate (NMDA) receptors [20], and by regulating gamma-aminobutyric acid (GABA) synapses [27]. Our study demonstrated that donepezil is able to activate PKC and MARCKS by increasing their phosphorylation, which may be the molecular basis for its effects on cell viability and neuroprotection.

MARCKS phosphorylation is regarded as a marker of PKC activation. In their inactivated states, PKC isoforms are localized in the cytosolic fraction, but they translocate to the membrane fraction after activation. To determine whether donepezil can affect the subcellular distribution of PKC isoforms, PKCa and PKCε, the two main isoforms involved in the pathogenesis of AD, were evaluated after donepezil treatment. Since STED analysis technology is not available in our lab, we performed further experiments to support our point of view. To detect the subcellular distribution of the PKC isoforms, immunocytochemical staining was performed, and the subcellular distribution of the PKCα and PKCε proteins in the cytosolic and membrane fractions was also measured by Western blotting, as in our previous study [28]. The results showed that donepezil decreased PKCα and PKCε expression in the cytosolic fraction but increased the expression of these two isoforms in the membrane fraction (Fig. 3). This implies that donepezil can induce the translocation of the PKCα and PKCε isoforms from the cytosolic fraction to the membrane fraction. Higher levels of the PKCα and PKCε isoforms were found in the membrane fraction in the donepezil-treated groups than in the vehicle-treated groups, supporting our conclusion that donepezil can activate the PKC signalling pathway. Activation of the PKC signalling pathway may thus become a key to the development of new drugs for the treatment of dementia in Alzheimer’s disease, which may indicate the clinical significance of our study.

Apoptosis and subsequent loss of neuronal function induced by various pathological and physiological factors may be the common pathological changes in neurodegenerative diseases, and protecting the function of the remaining neurons is one approach to treatment. As a specific and potent acetylcholinesterase inhibitor, donepezil is able to improve cognitive function in mild and moderate AD patients by increasing cholinergic nerve function and activating PKC to antagonize apoptosis. Donepezil can also increase the secretion of amyloid precursor protein α to support neurons and consequently exert its neuroprotective effect [13].

In our previous study, two cell lines were used in one experiment. When we investigated the effects of the protein kinase C activator TPPB on amyloid precursor protein (APP) processing, we used PC12 cells and SH-SY5Y^APP695^ cells, human neuroblastoma SH-SY5Y cells stably transfected with human wild-type APP695 cDNA [29]. The same cells were used to study PMS777, a new cholinesterase inhibitor with anti-platelet activated factor activity [30]. We used human neuroblastoma SK-N-SH cells and PC12 cells to study the effect of deprenyl on APP processing [28]. These three experiments showed similar results, including the effects of these agents on APP expression, sAPPα secretion, and Aβ secretion, in the two cell lines [28–30]. In neuroprotective studies, we investigated the effects of erythropoietin on Aβ-induced neurotoxicity in SH-SY5Y cells and PC 12 cells, and the results showed the same trend [31, 32]. Although the two cell lines come from two different species, the experiments showed similar results. Thus, in the present study, we exposed PC12 cells to Aβ as an in vitro model for investigating the neuroprotective effects of donepezil.

Our study is clinically significant because extracellular deposits of Aβ protein is able to result in formation of senile plaques in the hippocampus, which is the pathological characteristics of AD associated with neurodegeneration. Among the fragments studied thus far, Aβ_25-35_ represents the shortest fragment of Aβ, which is processed in vivo by brain proteases. Aβ_25-35_ is the functional domain of Aβ required for its neurotoxic effect in dementia clinically, and it retains the toxicity of the full-length peptide [33]. It is highly cytotoxic to neuronal cells and widely used both in vitro and in vivo in neuroscience research [34], including our own previous studies [2, 31, 32, 35]. Thus, Aβ_25-35_ was chosen for this experiment. The doses of Aβ_25-35_ in our study were used according to relevant studies [36, 37]. We firstly applied 5, 10, 20 or 50 μmol/L of Aβ_25-35_ to test the neurotoxicity of Aβ_25-35_ in the PC 12 cells. After this test, we chose the concentration of 20 μmol/L of Aβ_25-35_ for the protective experiment of donepezil. At the concentration of 20 μmol/L of Aβ_25-35_ to test the protective effect of donepezil, the cell viability was 56% (as determined by the MTT assay). If donepezil is added after Aβ administration, there may not be enough cells left to be rescued by donepezil, and the neuroprotective effect of donepezil would consequently be difficult to show. If a lower concentration of Aβ_25-35_ (5 or 10 μmol/L, for instance) is used, the neuroprotection afforded by donepezil may not be necessary, and thus its potential neuroprotective ability would also be difficult to show. Thus, we used Aβ_25-35_ at a concentration of 20 μmol/L (which damaged a portion of the cells) and added donepezil before Aβ administration to show its neuroprotective effect against Aβ.

Pretreating cells with donepezil before adding Aβ is a prophylactic approach that may not mimic the in vivo situation. Before treatment with Aβ_25-35_ in our study, the effect of donepezil on cell viability was tested with the concentration of 0.5, 1, 5, 10, 20 and 50 μmol/L, none of which had a negative effect on the PC 12 cells. Then, donepezil at 5, 10, 20 and 50 μmol/L was used to test its neuroprotective effect on the PC 12 cells after treatment with Aβ_25-35_. The protective effect of donepezil was dose dependent when used in the doses range of 5, 10, 20 and 50 μmol/L for the treatment of PC 12 cells. In the treatment of cells, we used the concentration μmol/L of the donepezil. When used in human for the treatment of AD, the dose of donepezil is mg/d and the highest dose of donepezil is 10 mg per day, which is probably to prevent possible side effects caused by larger-dose donepezil.

In conclusion, donepezil can antagonize Aβ_25-35_-induced neurotoxicity in PC 12 cells, and the activation of PKC may account for the neuroprotective effect of donepezil. Activation of PKC may become a key to develop novel approaches for the treatment of dementia in Alzheimer’s disease.

## Data Availability

All data and materials will be available from the corresponding author.
